# Benefits of focus group discussions beyond online surveys in course evaluations by medical students in the United States: a qualitative study

**DOI:** 10.3352/jeehp.2018.15.25

**Published:** 2018-10-16

**Authors:** Katharina Brandl, Soniya V. Rabadia, Alexander Chang, Jess Mandel

**Affiliations:** 1 Skaggs School of Pharmacy and Pharmaceutical Sciences, University of California San Diego, La Jolla, CA, USA; 2 School of Medicine, University of California San Diego, La Jolla, CA, USA; Hallym University, Korea

**Keywords:** Qualitative analysis, Program evaluation, Evaluation studies

## Abstract

In addition to online questionnaires, many medical schools use supplemental evaluation tools such as focus groups to evaluate their courses. Although some benefits of using focus groups in program evaluation have been described, it is unknown whether these inperson data collection methods provide sufficient additional information beyond online evaluations to justify them. In this study, we analyze recommendations gathered from student evaluation team (SET) focus group meetings and analyzed whether these items were captured in open-ended comments within the online evaluations. Our results indicate that online evaluations captured only 49% of the recommendations identified via SETs. Surveys to course directors identified that 74% of the recommendations exclusively identified via the SETs were implemented within their courses. Our results indicate that SET meetings provided information not easily captured in online evaluations and that these recommendations resulted in actual course changes.

The evaluation of medical school courses requires a range of methods to gain a sufficiently comprehensive view of the program [1,2]. Most medical schools use quantitative methods in the form of closedend rating scales with 1 or 2 opportunities for open-ended comments [3]. These quantitative methods are simple in design, easy to operate, and useful for obtaining information from a large number of students. However, the scope of online evaluations is limited and several studies have reported that students fill out these evaluations mindlessly [4]. As a consequence, some medical schools have implemented qualitative collection methods such as focus groups to supplement online course evaluations and to ‘tell the story’ behind closedended rating scales [5,6]. Focus groups provide space for clarifying questions and allow a face-to-face dialogue between students and faculty. In addition, focus groups can encourage student interactions that reveal issues not addressed in online evaluations and promote discussion of practical solutions. The process of organizing, conducting, and analyzing data from focus groups requires significant resources. It is unclear, however, whether these in-person qualitative methods provide sufficient additional information beyond online evaluations to warrant investment in them. Furthermore, any evaluation system must be judged on whether the results collected actually lead to curricular changes.

The University of California, San Diego (UCSD) School of Medicine has implemented student evaluation team (SET) focus group meetings for the evaluation of preclerkship courses in addition to online questionnaires [5]. In this study we analyzed the recommendations gathered in SET meetings and compared them to the information captured from the open-ended comments of online evaluations. We next determined whether recommendations from SET meetings resulted in actual course changes (Fig. 1).

SET meetings were scheduled after each of the preclerkship core courses. The course director, academic deans, and approximately 16 randomly selected students who recently completed the course participated. The Assistant Dean for Educational Development and Evaluation (Doctor of Philosophy in Psychology and Master in Health Profession Education), who is not involved the coursework, facilitated these meetings. In the meetings, students considered the course as a whole and commented on “what worked well in this course and what didn’t” [5].

Notes from 9 SET meetings for second-year medical student courses (academic year 2015–2016) taken by 2 second-year medical students (S.V.R. and A.C.) were analyzed. SET meetings were scheduled on 9/25/15 for course 1, 10/12/15 for course 2, 10/23/15 for course 3, 11/2/15 for course 4, 11/30/15 for course 5, 1/4/16 for course 6, 2/19/16 for course 7, 3/7/16 for course 8, and 3/18/16 for course 9, and lasted for 1 hour each. Feedback that included potential solutions was identified in a grounded theory-based approach and coded into the following 7 categories: issues related to specific teaching modalities used in courses, the overall course content, specific lectures (content and organization), sequencing of course events, administrative course components, exams, and study materials.

Open-ended comments from online questionnaires were analyzed for the same 9 preclerkship courses for second-year medical students. In these online questionnaires, a 20-item Likert-style survey was followed by a request for comments related to the course. The survey was administered after the end of each course and 714 deidentified responses from second-year medical students were collected. The overall response rate of the online questionnaires was 66%. A total of 293 comments from the online questionnaires of the 9 preclerkship courses were analyzed. Online comments corresponding to SET meeting comments were identified.

During the following year (2016–2017), surveys were sent to each course director (n= 9) as their course began. These surveys asked the course director whether they had implemented the suggested changes in their course. Course directors responded to each of the recommendations with “yes,” “somewhat,” or “no.” For the quantitative analysis of course directors’ responses, a response of “yes” for a specific recommendation was considered as 100% implemented. A response of “somewhat” was considered as 50% implemented, and a response of “no” as 0% implemented. Surveys were completed on 9/12/16 for course 1, 10/9/16 for course 2, 3/13/17 for course 3, 11/7/16 for course 4, 11/18/16 for course 5, 12/6/16 for course 6, 1/27/17 for course 7, 2/27/17 for course 8, and 2/27/17 for course 9. Raw data are available from Supplement 1.

## Ethic statement

The UCSD Institutional Review Board designated this study as an EBP/QI/QA (evidence-based practice, quality improvement, and quality assurance) project and therefore did not require full review (IRB approval no., 151319QI).

Analysis of the SET meeting notes yielded 69 suggested course improvements that included potential solutions, which were coded into the 7 categories listed earlier (Table 1). Of the 69 issues identified via SET, online evaluations captured 34 (49%). Specifically, SETs were superior in capturing feedback regarding specific teaching modalities in courses (18% appeared in online evaluations), problems related to the overall course content (25% online), and lecture content and organization (25% online). In contrast, online evaluations captured most of the deficiencies in study materials (80%), administrative course components (67%), exam-related problems (63%), and sequencing of course events (58%).

Survey data from the course directors identified that 74% of the recommendations captured exclusively in SETs (in contrast to online evaluations) translated into course changes (26 of 35). Table 1 lists all suggested improvements and indicates whether each item was implemented by the course director and captured in the open-ended comments from the online evaluations.

Evaluation is an integral part of medical education, and many tools are available to comprehensively characterize a program. One major purpose of collecting evaluations is to guide instructional improvement. Our analysis revealed that 74% of the SET-identified actionable items translated into course changes, implying that the focus groups served as a catalyst for discrete course adjustments. Studies have suggested that written comments may provide useful information that go beyond that of numerical ratings generated by closedended Likert-style questionnaires [7]. However, 2 major problems are associated with open-ended comments. First, interpreting students’ comments is not an easy task and there are no opportunities for asking clarifying questions. Second, open-ended comments often lack specificity and contextual factors [7]. Implementing a focus group as part of the evaluation process addresses both shortcomings. SET meetings facilitate negotiation, listening, and responding. Recommendations suggested by students in these meetings are discussed with faculty and deans in a collaborative dialogue. Students can explain proposed solutions and avoid confusion or misjudgments from faculty. In contrast to online open-ended comments, SET evaluations were rich in specific suggestions for improvement and also often included a contextual factor. Most importantly, our results indicate that suggestions identified in the SET meetings met the gold standard for evaluation comments—they actually led to course changes. The ‘give-and-take’ from multiple stakeholders in a course can best facilitate this process.

No single evaluation tool will capture the entirety of all potentially useful feedback. The choice of an evaluation model should no longer be a treasure hunt for the one perfect evaluation model. It should be viewed as an ‘all of the above’ approach, rather than a ‘best single answer’ choice. Our data indicate that including open-ended focus groups can provide rich solution-based feedback that makes this a worthwhile tool to add to the evaluation toolbox.

## Figures and Tables

**Fig. 1.**
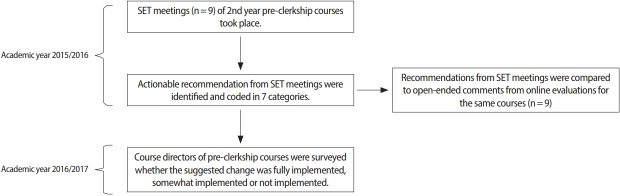
Flowchart of study design. SET, student evaluation team.

**Table 1.** Actionable recommendations (n=69) identified by SET input

Course	Actionable recommendations identified in SETs (by category)	Implementation^a)^	Captured in online evaluations^b)^
Study materials			
1	Implementing an additional practice quiz in a specific block.	Yes	No
1	Framing practice questions within clinical scenarios.	Yes	Yes
1	Improving the similarity of practice quiz questions and final exam questions.	Yes	Yes
3	Organizing practice quizzes by week regardless of the subject matter.	Yes	Yes
3	Including detailed explanations for the […] question on the practice quiz.	Yes	Yes
4	Increasing the number of practice quiz questions to ≥ 25.	Some	No
4	Providing practice quizzes as PDFs as well as on the online exam software.	No	Yes
4	Specifying the relevant lecture within each practice quiz question explanation.	Some	Yes
6	Increasing the resolution of specific images on the practice quizzes.	Yes	Yes
7	Adding more practice questions to a specific course syllabus.	Yes	Yes
Sequencing of events/coordinating events			
1	Adjusting the scheduling of a patient presentation that includes difficult psychosocial interactions and sensitive topics to allow adequate time for reflection.	No	No
1	Adjusting the schedule to provide any integrative reviews of a complex topic immediately after that topic’s presentation in lecture, rather than at the end of the course.	Some	Yes
2	Adjusting the schedule to provide a full day for processing between a complex topic presented in lecture and small group exercises on the same topic.	Yes	Yes
3	Improving coordination between the physical exam in the practice of medicine course with the corresponding material in the relevant block.	Yes	No
3	Ensuring that diseases presented in clinical skills sessions are described in lectures prior to those sessions.	Some	No
3	Adjusting the schedule to prevent the placement of a flipped lecture onto a lecture-heavy day, especially if the following day includes TBL on that material.	Some	Yes
4	Improving the scheduling of the laboratory sessions.	Yes	No
5	Adjusting the schedule to avoid 4-hour lecture blocks the day before the final exam.	Yes	Yes
6	Ensuring that conditions presented in case studies sessions are described in lectures prior to those sessions.	Yes	Yes
6	Adjusting the lecture schedule to avoid presenting particularly complex and difficult concepts immediately before the final exam.	Yes	Yes
8	Scheduling all pathology lectures after the relevant pathophysiology lectures.	Some	No
8	Adjusting the lecture schedule to reflect the topic sequence within the recommended textbook of the course.	Some	Yes
Course (administrative component)			
1	Posting of lecture notes beforehand to enable the students to download in time.	Yes	No
1	Clarifying the learning objectives on […].	Yes	Yes
1	Improving the clarity of acronyms used in lectures and proofreading lecture notes for typos.	Some	Yes
1	Clarifying specific details on the drug list posted on the course website.	Some	Yes
2	Encouraging lecturers to maintain timing of lectures to 50 minutes.	Yes	Yes
4	Improving the correlation of the drug list with the lecture material.	Yes	Yes
6	Clarifying the relative importance of textbook material early in the course.	Yes	Yes
6	Requiring lecturers to finish within their allotted time.	Yes	Yes
7	Adding space on small group handout to allow annotations.	Yes	No
7	Posting the drug list for […] on the course website.	Yes	No
8	Focusing reports of negative performance to students rather than including deans and other administrators.	Yes	No
8	Providing clear expectations on textbook reading (supplemental versus required).	Some	Yes
Course content			
1	Providing more emphasis for the importance of high-yield facts during the introduction lecture.	Yes	No
1	Adding an introductory lecture in a specific block.	Some	No
2	Adding definitions of […] to the lecture slides.	Yes	No
2	Include an integrative overview figure (favorite figure) to guide students to differentiate between different tests.	Yes	No
2	Adding organization to lectures and start with an important overview before adding details.	Some	Yes
2	Adding specific sessions within this course on how to write research papers.	Some	Yes
3	Adding the presentation of drugs into the […] lecture.	Some	No
4	Adding the discussion of specific diseases […] that are important for USMLE step 1.	Some	No
Exams			
2	Providing calculators on computerized exams other than the calculator embedded in the exam software.	Yes	No
3	Adding more images to a specific portion of the exam.	Yes	Yes
3	Adding explanations to the […] portion in the exam review session.	Yes	Yes
4	Providing images with higher resolution on the computerized exam.	Some	No
4	Matching the difficulty of practice questions with actual exam questions.	Yes	Yes
8	Modifying the questions to reflect USMLE guidelines for multiple choice questions and increasing the use of clinical vignettes in question stems.	Yes	Yes
8	Ensuring that images used on the computerized exam are high-resolution.	Some	Yes
9	For each exam question that requires multiple lab values or a common clinical vignette, ensure that each question provides the needed information.	Yes	No
Lecture organization/content (specific lectures)			
1	Increasing the emphasis of general concepts rather than small details in the […] lecture.	Yes	No
1	Matching the […] lecture content with the learning objectives.	Some	No
3	Adding more opportunities for interactive engagement and expanding on the pathophysiology of the […] lecture.	Yes	No
3	Improving the organization of the […] lecture.	Some	No
3	Eliminating duplicative material of the […] lecture.	No	Yes
4	Reducing the amount of slides in the […] lecture.	Yes	No
5	Reducing the research background in the […] lecture and increasing its clinical relevance.	Yes	No
8	Improving the organization for the […] lecture.	Yes	Yes
Specific teaching modalities			
1	Providing PowerPoint summary slides for the small groups to minimize the impact of facilitator variability between the groups.	No	No
2	Assigning a specific time in the beginning of the small group exercise for students to review the paper.	No	Yes
3	For in-class problem-solving sessions, posting detailed answers immediately after class.	Yes	No
3	Increasing the interactive component of the […] sessions.	Yes	No
4	Changing teaching modalities (small group activities should be replaced by TBL sessions).	Some	Yes
5	Eliminating slides that only show pathologic tissues, rather providing slides with additional information.	No	No
6	For case study problems, ensuring that each problem is formatted so it can be used later as a practice question, by providing the question with the answer on the following slide.	Yes	No
7	Improving facilitator training to ensure that proper etiquette is enforced in all small group sessions.	Yes	No
7	Improving facilitator training to ensure each facilitator provides an adequate overview of the disorder and associated pharmacology.	Yes	No
8	Improving the case vignettes during the laboratory sessions.	Some	No
9	Adding detailed explanations to the small group.	No	No

SET, student evaluation team; TBL, team-based learning; USMLE, United States Medical Licensing Examination.

a) Course directors revealed whether the suggested change was fully implemented (yes, n=41), somewhat implemented (some, n=21), or not implemented (no, n=7).

b) Actionable recommendations were captured (yes, n=34) or not captured (no, n=35) by open-ended comments from online evaluations.

## Supplementary materials

Supplement 1. Data files are available from https://doi.org/10.7910/DVN/ZFKN5R.

Supplement 2. Audio recording of the abstract.
